# Factors affecting intention to take COVID-19 vaccine among Pakistani University Students

**DOI:** 10.1371/journal.pone.0262305

**Published:** 2022-02-11

**Authors:** Mosharop Hossian, Md Abdullah Saeed Khan, Anum Nazir, Mohammad Hayatun Nabi, Mehedi Hasan, Ramisha Maliha, Mohammad Ali Hossain, Md Utba Rashid, Nizwa Itrat, Mohammad Delwer Hossain Hawlader

**Affiliations:** 1 Department of Public Health, North South University, Dhaka, Bangladesh; 2 Public Health Professional Development Society (PPDS), Dhaka, Bangladesh; 3 Pi Research Consultancy Center, Lalbagh, Dhaka, Bangladesh; 4 Department of Nutrition and Dietetics, The University of Faisalabad, Faisalabad, Punjab, Pakistan; 5 Ibn Sina Medical College Hospital, Kallyanpur, Dhaka, Bangladesh; 6 Nutrition and Clinical Services Division (NCSD), International Centre for Diarrhoeal Disease Research, Bangladesh (icddr,b), Mohakhali, Dhaka, Bangladesh; Marie Stopes International, PAKISTAN

## Abstract

**Background:**

Widespread vaccination coverage is essential for reducing the COVID-19 havoc and regarded as a crucial tool in restoring normal life on university campuses. Therefore, our research aimed to understand the intention to be vaccinated for COVID-19 among Pakistani university students.

**Methods:**

This cross-sectional study was conducted in five administrative units of Pakistan, i.e., Punjab, Sindh, Balochistan, Azad Jammu and Kashmir, and Khyber Pakhtunkhwa. We obtained data from 2,865 university students between 17^th^ January and 2^nd^ February, 2021, using a semi-structured and self-administered questionnaire. We used Stata (version 16.1, StataCorp LLC) for data management and analysis.

**Results:**

The majority (72.5%) of our respondents were willing to take COVID-19 vaccine. The current level of education had a statistically significant relationship with the intention to be vaccinated (*p* < 0.05). Respondents answered 11 questions encompassing five different domains of the Health Belief Model (HBM). All the items of HBM were significantly associated with the positive intention towards receiving the vaccine (*p* < 0.05). We conducted a multivariable logistic regression analysis to assess the relative contribution of different factors towards the intention to receive COVID-19 vaccine. Multiple factors such as belief that vaccination should be mandatory for everyone (AOR: 3.99, 95% CI: 3.20–4.98) and willingness to take vaccine even if it is not free (AOR: 3.91, 95% CI: 3.18–4.81) were observed to be associated with high odds of showing willingness to be vaccinated against COVID-19.

**Conclusion:**

Most of our study participants intended to take vaccines based on their belief regarding the high effectiveness of COVID-19 vaccine. But as rumor-mongers are generating and spreading conspiracy theories daily, the health department and policymakers need to undertake evidence-based campaigns through electronic and social media to ensure expected countrywide vaccination coverage. In this case, our study findings can serve as a foundation for them to ensure mass vaccination coverage among university students, which is crucial now to reopen the dormitories and restore everyday life on campuses.

## Introduction

The coronavirus disease-2019 (COVID-19) outbreak caused by the new severe acute respiratory syndrome-coronavirus-2 (SARS-CoV-2) virus is wreaking havoc throughout the world [1]. On January 30, 2020, the World Health Organization (WHO) identified the new COVID-19 as a worldwide pandemic and proclaimed an emergency [2]. The pandemic has resulted in a significant loss of human life across the globe and posed an unprecedented threat to public health, livelihood, and the workplace [3]. COVID-19 had afflicted 181,521,067 individuals globally as of June 2021, resulting in 3,937,437 fatalities [4], with 957,371 cases and 22,281 deaths documented in Pakistan [5]. Aside from disrupting the human life, the novel COVID-19 has dramatically slowed down the economies of China, United States of America, India, and Pakistan, as well as the entire world [6]. Therefore, to alleviate the community transmission, other than being quarantined and using personal protection equipment, everyone must be vaccinated, including people of Pakistan.

Researchers have been working feverishly on treatments to limit the COVID-19 pandemic. While particular highly effective antiviral medications have yet to be identified, significant progress has been made in developing vaccines to combat the disease [7]. Several vaccines, all promising in effectiveness and protection, were developed at a breakneck speed less than a year after the pandemic broke out [8]. Unfortunately, Pakistan has a history of vaccine hesitancy and refusal [9]. Vaccine hesitancy and rejection by the majority of the people are perhaps two of the most serious challenges for a successful vaccination program aimed at achieving herd immunity [10].

The Health Belief Model (HBM) is a prominent framework for analyzing human health behaviours. The HBM construct is made up of several subdomains, including perceived susceptibility, perceived severity, perceived benefits, perceived barriers, and cues to action [4]. Perceived susceptibility refers to a person’s perceptions of how vulnerable they are to infection, and perceived severity refers to the likelihood of changing their health-related behaviours to prevent potentially serious repercussions. In the context of vaccine acceptance, perceived benefits refer to a person’s thoughts about the desirable returns from getting vaccinated. In contrast, perceived barriers are beliefs that may limit a person’s willingness to be immunized against a specific disease. Extraneous elements that influence a particular health behaviour are known as cues to action. The HBM model has been employed as a key framework to analyze influenza vaccination acceptance behaviour in many different pieces of research [11, 12]. That is why assessing important HBM framework components that influence the intention to take the COVID-19 vaccines could be helpful in increasing vaccination coverage.

University students play a critical role in all societies. They are regarded as wise, influential, receptive, informed, and responsive to public health challenges. To ensure herd immunity and develop effective COVID-19 vaccination programs, it is crucial to identify the factors that influence the intention and behaviour of the specialized group with possible reluctance or hesitancy about COVID-19 vaccines. Furthermore, the majority of university students live in residential halls and shared housing. If the authority decides to reopen physical classes without enough vaccination coverage, the COVID situation will surely deteriorate. Considering the situation, Pakistan Government has taken a policy to vaccinate university students on a priority basis. [13]. Hence, it is critical to evaluate university students’ intentions regarding the COVID-19 vaccination, leading to more widespread knowledge distribution.

## Methodology

### Study design and participants

**Fig 1:**
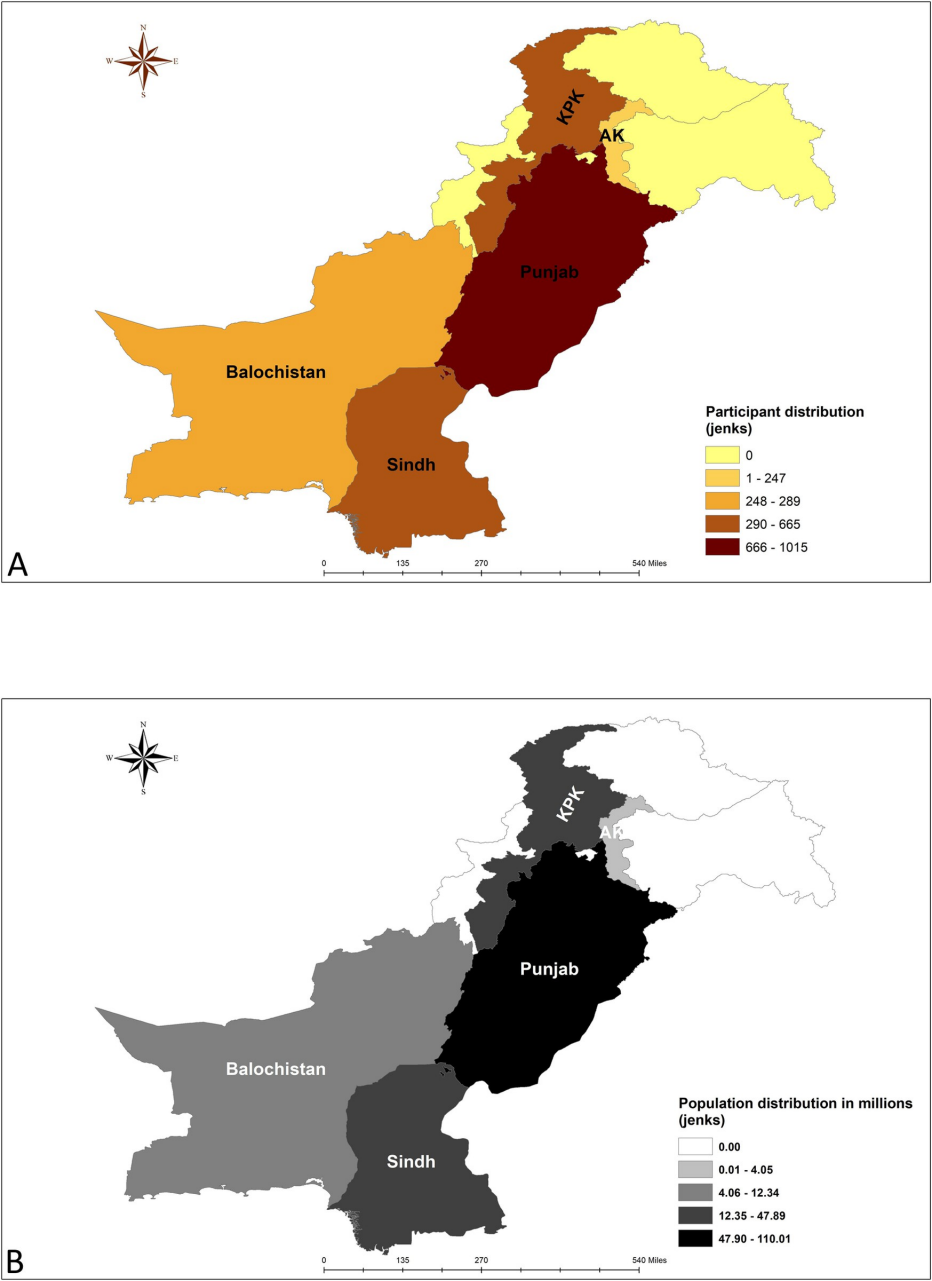
Administrative unit wise distribution of the study participants (A) and population (B). [Islamabad and Federally Administered Tribal Areas (FATA) were excluded] (Created with ArcGIS version 10.5).

This cross-sectional study was conducted among Pakistani university level students. The data collection period was between 17^th^ January and 2^nd^ February, 2021. All persons identifying as students of different universities were conveniently selected for study. As no study were available on the acceptance rate of COVID-19 vaccine among students, assuming an acceptance rate of 50% (p), and considering a standard normal variate of 1.96 (z) and a margin of error of 3% (d) we calculated the sample size (n) to be 1067 (using the formula n = z^2^pq/d^2^). As we intended to take participants from the main provinces along with Azad Kashmir (AK), we multiplied the estimate with 2 to account for the design effect and added 10% additional samples to account for the anticipated refusals. Hence the final sample size was 2348. Taking the distribution of population in different province (including AK) into account, the province-wise breakdown of intended sample size was 1219 from Panjab, 531 from Sindh, 338 from Khyber Pakhtunkhwa (KP), 137 from Balochistan, 55 from Federally Administered Tribal Areas (FATA), 45 from AK and 22 from Islamabad. But we intended to take as many samples as possible within the data collection period. Finally, a total of 2865 university students completed the survey; among them, 1015 were from Punjab, 665 from KP, 649 were from Sindh, 289 were from Balochistan, and 247 were from AK. We excluded those who were not interested or willing to give consent to participate in the study, and Islamabad and FATA could be not reached (Fig 1).

### Study procedures

We reached undergraduate and graduate level university students from five administrative units of Pakistan, i.e., Punjab, Sindh, Balochistan, AJK, and KP. A team of experienced researchers planned, executed and monitored the entire procedure. As we have used convenience sampling method, the data collectors approached as many potential respondents as possible irrespective of their socio-economic condition, and ethnicity to represent participants from different backgrounds. We informed the participants regarding the goals and objectives of the study, and explicit informed written consent was obtained from all study subjects. After that, we shared a pre-tested semi-structured and self-administered questionnaire with the respondents to write their responses. Our volunteers helped the participants through proper explanation when they faced difficulties to grasp specific item(s) while answering the questionnaire.

### Measures

The questionnaire we used in this study consisted of several sections: (a) Socio-demographic variables; (b) Impact and vulnerabilities of COVID-19; (c) Health belief model (HBM) variables; (d) Intention to receive COVID-19 vaccine. Overall, there were 26 items in the questionnaire and took around 7–8 minutes to complete. Our questionnaire was prepared based on the tool developed by Sherman et al. 2020 [14] in line with health belief model proposed by Hochbaum et al. 1950 [15].

The questionnaire was adapted by forward and backward translation, iterative revision, and consensus by experts. Each translation was reviewed by three independent health-care providers, psychologists and sociologists who were proficient in both English and Urdu. The accuracy of the translations was certified. There was cent percent strong agreement about the items in questionnaire among these subject experts.

The predictor variables were categorized into three blocks:

- The socio-demographic variables were: gender, marital status, education level, being involved in health care, area of primary residence (rural, semi-urban, urban), monthly family income, number of family members.
- Impact and vulnerabilities of COVID-19 related predictors were: previously being diagnosed as having COVID-19, any family member impacted by COVID-19, presence of senior citizen(s) in the family, chronic disease history (any or more than one of the following: hypertension, chronic kidney disease, Chronic obstructive pulmonary disease, chronic liver disease, etc.), previous vaccination history.
- The section—attitude and beliefs about COVID-19 and its vaccine, were developed based on the five domains of HBM model: perceived susceptibility, perceived severity, perceived benefits, perceived barriers and cues to action.

Items in the impact and vulnerabilities of COVID-19 and HBM model were measured on dichotomous scale (yes, no).

The outcome variable was the intention to take COVID-19 vaccine. It was measured by only one question—"When a coronavirus vaccination becomes available to you, are you going to take one?". The response was binary (yes–positive intention towards vaccine acceptance, no–hesitant/not intended to take vaccine).

Before widely disseminating the questionnaire to our participants, we conducted pre-testing of the questionnaire on randomly selected university level students who were kept excluded from original study. For the pre-testing, we enrolled university students from different socioeconomic classes. The authors took into account the suggestions made by participants and adjusted them, keeping things consistent with current literature. Then, the questionnaire was finalized after a thorough discussion among the authors and was then distributed to the study participants. We have shared the final questionnaire as S1 Questionnaire.

### Statistical analysis

We conducted descriptive analysis to present positive attitude of the participants towards COVID-19 vaccine acceptance in terms of socio-demographic characteristics, impact and vulnerabilities of COVID-19, past experience of vaccination and components of HBM. Chi-square tests of association was used to assess the relationship between categorical variables. Overall, intention towards vaccine acceptance was portrayed using pie chart. We performed multivariable logistic regression analysis whereby figuring out the influencing factors for COVID-19 vaccine taking intention. A receiver operating characteristics (ROC) curve was illustrated as an indicator of good fit of multivariable logistic regression model. Data processing and analysis was carried out using Stata (version 16.1, StataCorp LLC). All statistical tests were two-tailed and p-values less than 5% was considered as statistically significant.

### Ethical consideration

The study was approved by the Institutional Review Board (IRB)/Ethical Review Committee (ERC) of North South University (2021/OR-NSU/IRB-No.0304) as part of a multi-country study. Ethical approval for this study was also obtained from the Institutional Review Board (IRB) of Government College University Faisalabad (GCUF), Pakistan (GCUF/IRB/688). All the study procedures were carried out following the guidelines shared by the IRBs. We have followed the ethical standards outlined in the Declaration of Helsinki (1964) and its subsequent amendments or equivalent ethical principles.

## Results

**Fig 2:**
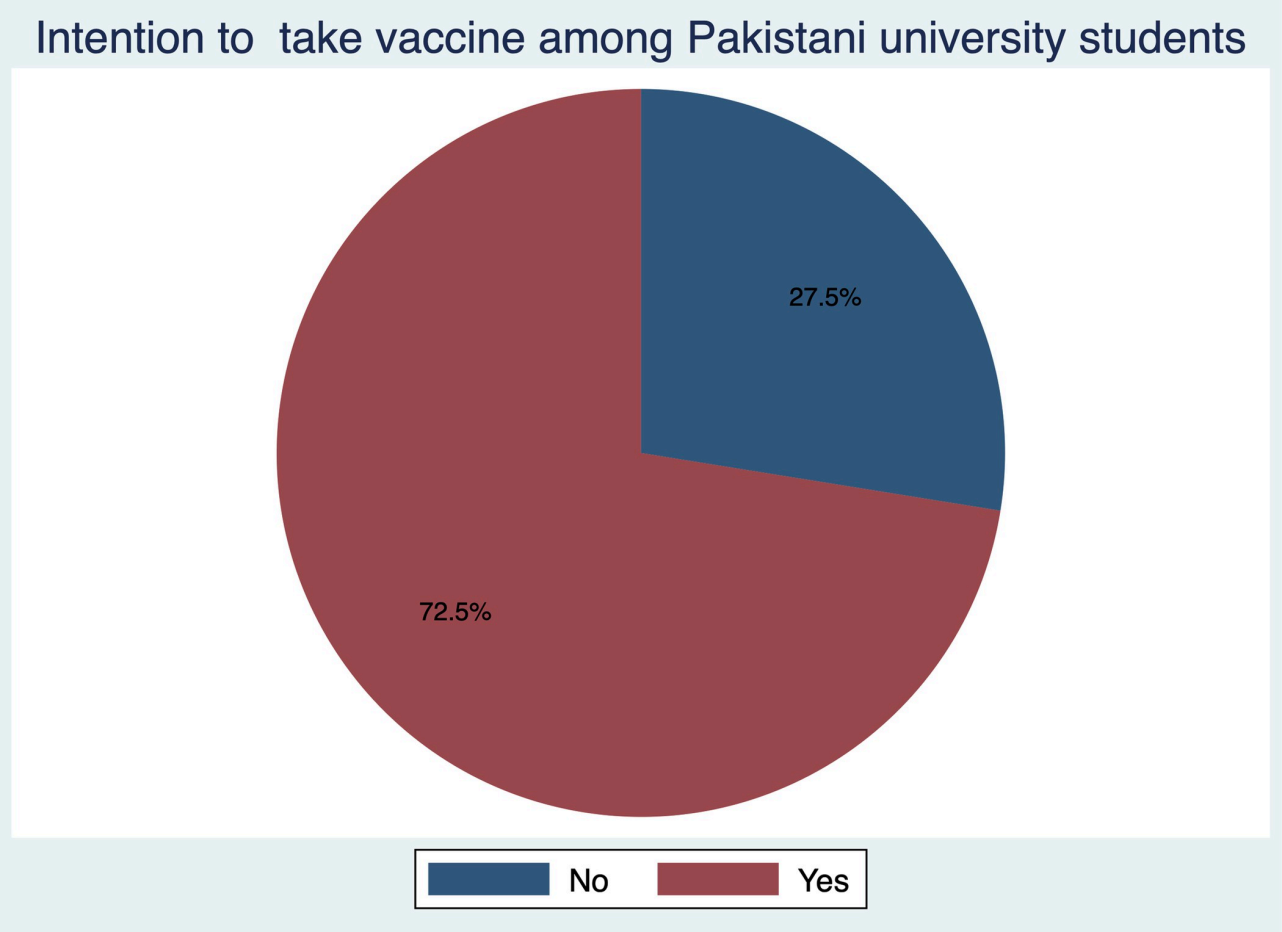
Distribution of respondents according to their intention to take vaccine.

A total of 2,865 respondents participated in the study. We received participation from respondents of various socio-demographic backgrounds by monthly family income types and area of primary residence. Overall, 72.5% of students (n = 2076) had positive intention and the remaining 27.5% (n = 789) had negative intention to take vaccine when it becomes available (Fig 2).

Majority of female (72.8%) and male (71.6%) participants intended to take the vaccine. The willingness to get vaccinated against COVID-19 was higher among married (74.4%) and unmarried (72.6%) respondents compared to divorced/widowed/separated respondents (52.0%). A substantial portion (70.8%) of the undergraduate-level students showed interest to take COVID-19 vaccine, while the intention was even higher among graduate-level students (75.6%). Majority (74.4%) of the students involved with health care had the intention to take the vaccine. Similar proportion of participants from small (72.7%) and large (72.3%) families expressed their intention to get vaccinated. However, after conducting chi-square test of association, only education level was found statistically significantly associated with intention to take COVID-19 vaccine (p<0.05) (Table 1).

**Table 1:** Socio-demographic characteristics of the study participants (n = 2865).

		Intention to take COVID-19 vaccine		*p*-value
	Total †	Yes ‡	No ‡	
Variables	n	n (%)	n (%)	
**Sex**				
Male	644	461 (71.6)	183 (28.4)	0.376
Female	2201	1603 (72.8)	598 (27.2)	
3rd Gender	20	12 (60.0)	8 (40.0)	
**Marital Status**				
Married	86	64 (74.4)	22 (25.6)	0.066
Unmarried	2754	1999 (72.6)	755 (27.4)	
Divorced/Widowed/ Separated	25	13 (52.0)	12 (48.0)	
**Current Level of Education**				
Undergraduate Student	1866	1321 (70.8)	545 (29.2)	0.006
Graduate Student	999	755 (75.6)	244 (24.4)	
**Health Care Worker**				
No	2259	1625 (71.9)	634 (28.1)	0.223
Yes	606	451 (74.4)	155 (25.6)	
**Monthly Family Income (PKR)**				
≤ 20000	1953	1424 (72.9)	529 (27.1)	0.711
21000 to 40000	275	198 (72.0)	77 (28.0)	
≥ 41000	637	454 (71.3)	183 (28.7)	
**Primary Residence**				
Rural	414	298 (72.0)	116 (28.0)	0.888
Semi-urban	2178	1577 (72.4)	601 (27.6)	
Urban	273	201 (73.6)	72 (26.4)	
**Family Type**				
Small (⩽ 5)	1153	838 (72.7)	315 (27.3)	0.829
Large (≥ 6)	1712	1238 (72.3)	474 (27.7)	

**Note:** *p*-values were determined by Chi-square tests

† Data expressed as **n** within rows

‡ Data expressed as **n (%)** within rows.

The perceived impact of COVID-19 influenced the intention to take vaccine against it (Table 2). Majority (68.8%) of participants affected from COVID-19 were willing to take the vaccine. In case of family members affected by COVID-19, almost 8 out of every 10 participants (78.0%) were ready to be vaccinated. We observed similar intentions among families having any elderly member(s) (> 60 years); more than three-quarters of them agreeing to take the vaccine. A similar trend was also observed in the participants having chronic disease(s) (78.8%) and vaccinated for any other disease(s) in the last few years (79.9%). After conducting chi-square test, we found—family members affected by COVID-19, having any elderly member (> 60 years) in the family and previous vaccination history were significantly associated with willingness to take COVID-19 vaccine (*p* < 0.05).

**Table 2:** Impact and vulnerabilities of COVID-19, past experience of vaccination and their association with the intention to take COVID-19 vaccine.

		Intention to Take COVID-19 Vaccine		
	Total †	Yes ‡	No ‡	
Questions on impact and vulnerabilities	n	n (%)	n (%)	*p*-value
**Were you diagnosed as having COVID-19?**				
No	2618	1906 (72.8)	712 (27.2)	0.181
Yes	247	170 (68.8)	77 (31.2)	
**Was any of your family members affected by COVID-19?**				
No	2292	1629 (71.1)	663 (28.9)	0.001
Yes	573	447 (78.0)	126 (22.0)	
**Do you have any elderly (>60 years) member in the family?**				
No	1518	1058 (69.7)	460 (30.3)	<0.001
Yes	1347	1018 (75.6)	329 (24.4)	
**Do you have any chronic disease?**				
No	2747	1983 (72.2)	764 (27.8)	0.115
Yes	118	93 (78.8)	25 (21.2)	
**Did you take any vaccine within the last few years?**				
No	2427	1726 (71.1)	701 (28.9)	<0.001
Yes	438	350 (79.9)	88 (20.1)	

**Note:** *p*-values were determined by Chi-square tests

† Data expressed as **n** within rows

‡ Data expressed as **n (%)** within rows.

Participants were asked 11 questions encompassing five domains of the HBM. All the items of different domains of the HBM were significantly associated with COVID-19 vaccine-taking intention (*p* < 0.05). Most of the participants (74.2%) were worried about catching the coronavirus, and 54.8% of the study subjects (n = 1571) believed that they were not immune to COVID-19. In the perceived severity domain, we observed that most respondents thought coronavirus would be a mild illness for them, and too much fuss was being made about the risk of the coronavirus, although, 81.2% of respondents (n = 2325) reported that the pandemic significantly impacted their lives. Under the perceived benefit domain, more than eight-tenths (n = 2362) of the participants had a strong belief regarding the effectiveness of COVID-19 vaccine, and nearly three-fourth (n = 2195; 76.6%) of the participants said vaccination should be mandatory for everyone. In contrast, 74.1% (n = 2124) of the total participants reported they were worried about the vaccine’s side effects. Although nearly 90% (n = 2507, 87.5%) of the respondents believed all of them were responsible for reducing the spread of COVID-19, only 1753 participants (61.2%) were willing to pay for the vaccine (Table 3).

**Table 3:** Beliefs and attitude towards COVID-19 and its vaccine in relation to intention to take COVID-19 vaccine.

		Intention to Take COVID-19 Vaccine		
	Total †	Yes ‡	No ‡	
Questions on belief and attitude	n	n (%)	n (%)	*p*-value
**Perceived Susceptibility**				
**Are you worried about catching coronavirus?**				
No	740	408 (55.1)	332 (44.9)	<0.001
Yes	2125	1668 (78.5)	457 (21.5)	
**Do you believe you are immune to the Coronavirus?**				
No	1571	1171 (74.5)	400 (25.5)	0.006
Yes	1294	905 (69.9)	389 (30.1)	
**Perceived Severity**				
**Do you believe that the Coronavirus disease would be a mild illness for you?**				
No	1347	924 (68.6)	423 (31.4)	<0.001
Yes	1518	1152 (75.9)	366 (24.1)	
**Do you think too much fuss is being made about the risk of the Coronavirus?**				
No	1178	814 (69.1)	364 (30.9)	0.001
Yes	1687	1262 (74.8)	425 (25.2)	
**Did the Coronavirus pandemic have a big impact on your life?**				
No	540	299 (55.4)	241 (44.6)	<0.001
Yes	2325	1777 (76.4)	548 (23.6)	
**Perceived Benefits**				
**Do you think vaccination should be made mandatory for everyone?**				
No	670	253 (37.8)	417 (62.2)	<0.001
Yes	2195	1823 (83.0)	372 (17.0)	
**Do you think people who are at risk of serious illness from the Coronavirus, need to be vaccinated on priority basis?**				
No	1043	790 (75.7)	253 (24.6)	0.003
Yes	1822	1286 (70.6)	536 (29.4)	
**Do you think vaccines will work against COVID-19?**				
No	503	180 (35.8)	323 (64.2)	<0.001
Yes	2362	1896 (80.3)	466 (19.7)	
**Perceived Barriers**				
**Are you afraid/concerned about the safety/side effects of the COVID-19 vaccine?**				
No	741	457 (61.7)	284 (38.3)	<0.001
Yes	2124	1619 (76.2)	505 (23.8)	
**Cues to Action**				
**Do you think we are all responsible for reducing the spread of the Coronavirus?**				
No	358	180 (50.3)	178 (49.7)	<0.001
Yes	2507	1896 (75.6)	611 (24.4)	
**Do you agree to take the COVID-19 vaccine if it is not free?**				
No	1112	549 (49.4)	563 (50.6)	<0.001
Yes	1753	1527 (87.1)	226 (12.9)	

**Note:** *p*-values were determined by Chi-square tests

† Data expressed as **n** within rows

‡ Data expressed as **n (%)** within rows.

Table 4 represents the simple and multivariable logistic regression analyses of factors associated with the positive intention to take COVID-19 vaccine. The thought that vaccination should be mandatory for everyone (Adjusted Odds Ratio [AOR]: 3.99, 95% Confidence Interval [CI]: 3.20–4.98) and willingness to take vaccine even if it is not free (AOR: 3.91, 95% CI: 3.18–4.81) were found to have the highest odds of having positive intention to be vaccinated against COVID-19. Participants who were anxious about catching coronavirus had greater odds (AOR: 1.59, 95% CI: 1.26–2.01) of willingness to take the vaccine. Those who held belief regarding the high effectiveness of COVID-19 vaccine were more willing to get vaccination (AOR: 2.97, 95% CI: 2.31–3.83). Participants who said “only people who were at risk of serious illness from coronavirus, need to be vaccinated on a priority basis” were 44% less likely to take COVID-19 vaccine (AOR: 0.56, 95% CI: 0.45–0.71). Participants who believed everyone has a responsibility to reduce the spread of COVID-19 were found to be 1.54 times (AOR: 1.54, 95% CI: 1.14–2.08) more likely to be vaccinated against COVID-19. Lastly, graduate-level students had higher odds of COVID-19 vaccine-taking intention (AOR: 1.30, 95% CI: 1.05–1.61) than undergraduate-level students.

**Table 4:** Sociodemographic factors, and health-beliefs regarding COVID-19 association with vaccine acceptance.

Variables	Univariate Analysis	Multivariable Analysis
	OR (95% CI)	AOR (95% CI)
**Current Level of Education**		
Undergraduate Student	1	1
Graduate Student	1.28 (1.07–1.52)*	1.30 (1.05–1.61)*
**Was any of your family members affected by COVID-19?**		
No	1	1
Yes	1.44 (1.16–1.79)*	1.15 (0.87–1.50)
**Do you have any members in your family who are over 60 years old?**		
No	1	1
Yes	1.35 (1.14–1.59)**	1.15 (0.94–1.42)
**Did you take any vaccine within the last few years (willingly/out of need)?**		
No	1	1
Yes	1.62 (1.26–2.07)**	1.22 (0.90–1.65)
**Perceived Susceptibility**		
**Are you worried about catching coronavirus?**		
No	1	1
Yes	2.97 (2.49–3.55)**	1.59 (1.26–2.01)**
**Do you believe you are immune to the Coronavirus?**		
No	1	1
Yes	0.79 (0.67–0.94)*	1.00 (0.81–1.22)
**Perceived Severity**		
**Do you believe that the Coronavirus disease would be a mild illness for you?**		
No	1	1
Yes	1.44 (1.22–1.70)**	1.10 (0.90–1.36)
**Do you think too much fuss is being made about the risk of the Coronavirus?**		
No	1	1
Yes	1.33 (1.13–1.57)*	1.02 (0.82–1.25)
**Did the Coronavirus pandemic have a big impact on your life?**		
No	1	1
Yes	2.61 (2.15–3.18)**	1.24 (0.95–1.60)
**Perceived Benefits**		
**Do you think vaccination should be made mandatory for everyone?**		
No	1	1
Yes	8.08 (6.67–9.79)**	3.99 (3.20–4.98)**
**Do you think people who are at risk of serious illness from the Coronavirus, need to be vaccinated priority basis?**		
No	1	1
Yes	0.77 (0.65–0.91)*	0.56 (0.45–0.71)**
**Do you think vaccines will work against the COVID-19?**		
No	1	1
Yes	7.30 (5.93–8.99)**	2.97 (2.31–3.83)**
**Perceived Barriers**		
**Are you afraid/concerned about the safety/side effects of the COVID-19 vaccine?**		
No	1	1
Yes	1.99 (1.67–2.38)**	1.04 (0.82–1.33)
**Cues to Action**		
**Do you think we are all responsible for reducing the spread of the Coronavirus?**		
No	1	1
Yes	3.07 (2.45–3.85)**	1.54 (1.14–2.08)*
**Do you agree to take the COVID-19 vaccine if it is not free?**		
No	1	1
Yes	6.93 (5.77–8.32)**	3.91 (3.18–4.81)**

Note

*OR = Odds ratio; AOR = Adjusted odds ratio; CI = Confidence Interval; *p*-value significant at *<0.05 and

**<0.001.

**Fig 3:**
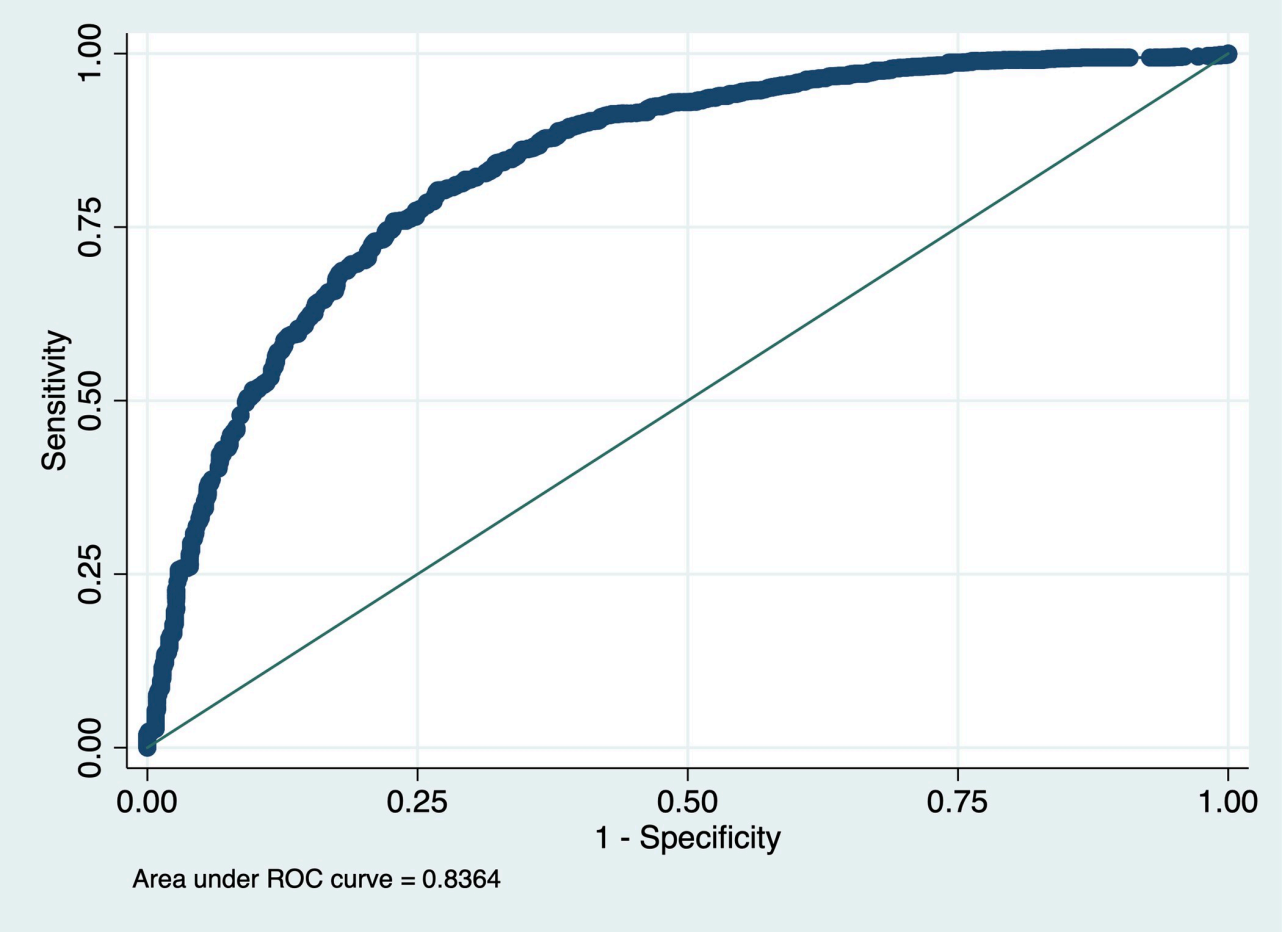
Receiver Operating Characteristic (ROC) analysis of the multivariable logistic regression model.

Area under the ROC curve shows that the multivariable logistic regression model was 83.6% good fit (Fig 3). The area under ROC curve was determined by plotting sensitivity versus 1- specificity. Our model could correctly classify 83.6% of the positive and negative intentions to get the COVID-19 vaccine.

## Discussion

Intention is an elemental factor which influences the acceptance of health behaviours [16]. And as for vaccination the actual vaccine uptake is bound to be lesser than the intention [17]. Hence, it is imperative to address the factors that influence the intention to vaccinate in order to support policy, rapid vaccination coverage and greater outcome. In this study the HBM was used to appraise the factors influencing the intention of receiving COVID-19 vaccine. This model has been used in multiple studies to identify the factors affecting the Influenza vaccination uptake [18, 19]. To increase the positive intention of the vaccination, reconnoitering HBM components that enhance COVID-19 vaccination might be a crucial step.

We found that overall, 72.5% of the students in this study had positive intention towards vaccination which is similar to that found among general population. One European study published similar result where 73.9% of the participants were willing to receive the vaccine [20] with 71.7% positive intention in the UK [21]. In a global survey of 19 countries, 71.5% participants were found to have positive intention to vaccinate [22]. Almost an equal portion of male and female participants were found to be willing to be vaccinated in this study. However, the ratio varied from study to study with some studies claiming to observe a higher hesitancy among women [23–25], while other reporting the opposite [22], or nearly equal ratios [26–28]. This could translate as existence of balanced gender-based public health policies and communication strategies [29]. Interestingly, graduate level students were found to be more positive towards getting a COVID-19 vaccine than under-graduate students indicating a maturation of thought among elder students. As educated people tend to look for and accept scientifically established facts far easily than people with less or no education, indicating rumors and conspiracy theories have little to no influence on the more educated ones [30]. Numerous similarities can be observed across the literature [31–35].

In the perceived susceptibility domain, almost one fourth (74.2%, n = 2125) of our participants were worried about being infected by the coronavirus. This represents a high level of susceptibility perception which could spring preventive measures in this outbreak aiding in a greater epidemic control. Participants who were worried about catching coronavirus disease were more likely to show the intention to get vaccinated. This heightened sense of risk perception about COVID-19 influencing the vaccination intention has been reported by other researchers. It has been found that low or nonexistent risk perception of being affected by COVID-19 sways the intention to get vaccinated in the downward direction [25, 27, 28, 36, 37]. However, 54.8% (n = 1571) believed that they are not immune to COVID-19 warranting measures to increase risk perception.

In perceived severity domain, it was observed that four-fifths of the respondents agreed when asked if the pandemic had a big impact on their lives. But the perception regarding severity of COVID-19 was less tuned with around half of the participants thinking coronavirus disease to be a mild one and a little more than half of the participants thinking that too much fuss was being made about the disease. Paradoxically this thinking didn’t negatively affect their intention to take vaccine contrary to what was suggested in previous studies [38, 39]. The overwhelming impact of the COVID-19 in peoples’ life might be instrumental in producing such conundrums. This is also supported by the observation that our participants were more likely to take the vaccine if they had a family member who was infected by COVID-19 or was vulnerable (e.g., elderly). In addition, accumulating evidences on relatively severe illness among elderly comorbid people by the coronavirus [40, 41] might be important determinants of such motivations.

Quite expectedly we have seen that a positive intention to take vaccine was associated with a positive benefit perception among the participants. Eighty two percent of the respondents perceived that the vaccine will be effective, 76.6% thought that the vaccine should be mandatory for everyone, and more than three fifth opted for vaccination on a priority basis. These findings are in conformity with the HBM as it predicts that people with positive attitudes and strong subjective norms are more likely to accept a vaccine [39].

The barriers to the willingness of receiving vaccine surfacing in our study were also apparent in previous studies [20, 25, 26, 42, 43]. Seventy four percent participants conveyed their concern about the safety and side effects of the COVID-19 vaccine. However, this appears not to have influenced the intention to take vaccine among the students. It is probably because, denial of COVID-19 vaccination has lessened after information on its safety and efficacy were being disseminated. This also proves how intention and hesitancy are complex constructs that are affected by several factors [44, 45].

In the cues to action domain, we observed that those who believed that everyone is responsible for reducing the spread of coronavirus disease and those who were willing to pay for the vaccine if it is free were significantly more willing to take a vaccine upon availability than their counterparts. However, only 61.2% of our study participants were willing to pay for the vaccine. This percentage is much higher in studies conducted in Indonesia and Ecuador. They concluded that an estimated 78.3% and 85.0% of the population respectively, are willing to pay for the vaccine; majority of the Australian citizens are willing to pay to reduce the waiting time [28, 46, 47]. Complex regional and personal economic and social factors come into play behind such decisions and might be working behind the thoughts of our participants.

According to the HBM and the theory of Planned Behavior, people with a belief that they have a high vulnerability to be ill are more willing to accept a vaccine [38]. But our observation suggests that family cues and a sense of belonging are also crucial in defining perception of vulnerability among students. Although majority of the students, who were previously diagnosed as COVID-19 positive, had intention of taking the vaccine, the negative physical, psychological and social impacts of COVID-19 might also have been in play. A study conducted in Bangladesh stated that individuals already diagnosed as COVID-19 positive are three times more intent than the unaffected populace [48]. Other studies from Saudi Arabia and France also mentioned observations that are in line with these findings [49, 50]. Additionally, more than three quarters of participants from families having any elderly (aged more than 60 years) member and 78.0% of those who had family members affected by COVID-19 were highly likely to get vaccinated. Social responsibility, negative impacts of COVID-19, higher education and trust on health care system along with positive experience with vaccination might be the governing factors here [51].

Another important determinant is the previous experience of vaccination. We found past history of vaccination to be significantly associated with COVID-19 vaccination intention. Around Eighty percent of the respondents that previously got vaccinated for any disease intended to take COVID-19 Vaccine, which was also reflected in the result of Several U.S. and Chinese studies with similar conclusion [25, 31, 43, 49]. Another important factor is the presence of a chronic disease. We noted that 78.8% of the respondents having a chronic disease were planning on getting COVID-19 vaccine. This scenario was similar in UK, Ireland and Bangladesh [23, 52]. As people with chronic disease consider themselves included in the more vulnerable group, their higher willingness is justified and consistent with the predictions of HBM. It may also boost greater COVID-19 awareness and greater communication and access to information in the community.

This study, like most, is burdened by some limitations. As the data collection was according to convenience owing to our incapability of covering all the provinces, lack of generalizability may prevail. Apart from the selection bias due to convenient sampling, we used a single item measurement of vaccine intention and thus are not exempted from potential bias. As vaccine intention and hesitancy are complex and multidimensional constructs, diverse types of data usage and multiple measurement approaches are suggested to precisely pinpoint the factors of influence. The strength of this study is the large educated sample size and usage of the HBM in data collection. The main strength of the HBM is that it is easy to implement, apply, and test because of its use of simplified health-related constructs and thus it increases the reliability [53, 54].

## Conclusion

Our study explored the factors affecting intention to take COVID-19 vaccine among Pakistani University Students where the current level of education had a statistically significant relationship with the intention to be vaccinated. The majority of our study participants intended to take vaccines based on their belief regarding the high effectiveness of the COVID-19 vaccine. We also found that most of the items of HBM were significantly associated with the positive intention towards receiving the COVID-19 vaccine. But as a group of people are generating and spreading conspiracy theories daily, the health department and the policymakers need to undertake evidence-based campaigns through all means to ensure expected countrywide vaccination coverage. In this case, our study findings can serve as a foundation for them to ensure mass vaccination coverage among university students, which is crucial now to restart offline classes and restore normal life on campuses.

## Supporting information

S1 Dataset (DTA)

S1 Questionnaire (DOCX)
