# Supplementary material for: Factors affecting intention to take COVID-19 vaccine among Pakistani University Students
Source: PLoS One. 2022 Feb 11;17(2):e0262305. doi: 10.1371/journal.pone.0262305 (PMC8836301; doi:10.1371/journal.pone.0262305)
Supplement: S1 Questionnaire — (DOCX) [file pone.0262305.s002.docx]

Survey on Belief, Attitude, and Intention About COVID-19 Vaccine Among University Students

# General information:

- ID (to be given by the authority):
- Date of interview:
- Name of the respondent (Optional):

- Current address:
  - District:
  - Division/Province:

1. Did you previously participate in this survey?
   1. Yes, I did B. No, I didn’t

(If you participated in this survey previously, please do not proceed further, and submit the form)

# Socio-demographic Informa- tion:

1. Sex:
   1. Male B. Female C. Third Gender
2. Marital Status:
   1. Married
   2. Unmarried
   3. Divorced
   4. Widowed
   5. Separated
3. Level of Education:
   1. Undergraduate level student
   2. Graduate level student
4. Are you involved in health care?
   1. Yes B. No
5. Monthly family income (in PKR):

A. *<*10000

B. 10000 to 20000

C. 20001 to 30000

D. 30001 to 40000

E. 40001 to 50000

F. 50001 to 60000

G. *>*60000

1. Total number of family members:
2. Residence:

- Rural
- Urban
  - Semi-urban
  - City

# Impact of COVID-19

1. Were you diagnosed as having COVID-19?
   1. Yes B. No
2. Were any of your family members affected by COVID-19?
   1. Yes B. No
3. Do you have any chronic disease (e.g., DM, HTN, CKD, COPD, CLD, or any other chronic diseases)?

? A. Yes B. No

1. Do you have any members in your family who are over 60 years old?
   1. Yes B. No

**Please go to the next page**⇒

1

# Previous Vaccination

1. Did you take any vaccine within the last few years (willingly/out of need)?
   1. Yes B. No
2. If the answer is ‘no’, why not? (multiple responses can be given)
   1. I am afraid of the needle
   2. Vaccine is costly
   3. I didn’t need a vaccine
   4. A vaccine has side effects
   5. I don’t know where to get a vaccine
   6. I don’t know about vaccine
   7. Others (Please specify):

# Attitudes and beliefs about COVID-19

1. Are you worried about catching the Coro- navirus?
   1. Yes B. No
2. Do you believe that the Coronavirus dis- ease would be a mild illness for you?
   1. Yes B. No
3. Do you think too much fuss is being made about the risk of the Coronavirus?
   1. Yes B. No
4. Do you think we are all responsible for re-ducing the spread of the Coronavirus?
   1. Yes B. No
5. Do you believe you are immune to the Coronavirus?
   1. Yes B. No
6. Did the Coronavirus pandemic have a big impact on your life?
   1. Yes B. No

# Attitude and beliefs about COVID-19 vaccine

1. Do you think vaccination should be made mandatory for everyone?
   1. Yes B. No
2. Do you think people who are at a risk of serious illness from the Coronavirus, need to be vaccinated on priority basis?
   1. Yes B. No
3. Are you afraid/concerned about the safety/side effects of COVID-19 vac- cine?
   1. Yes B. No
4. Do you agree to take the COVID-19 vac- cine if it is not free?
   1. Yes B. No
5. Do you think vaccines will work against the COVID-19?
   1. Yes B. No

# Intention to take COVID-19 vaccine

1. When a Coronavirus vaccination becomes available to you, are you going to take one?
   1. Yes B. No
2. Please specify why you are not willing to take the vaccine? (multiple responses can be given)
   1. I am afraid of the needle
   2. I am not sure if the vaccine will be free or not
   3. The vaccine might have side ef- fects/safety concerns
   4. I don’t know for how many days I can get protection by getting the vaccine
   5. I have doubts about the proper preser- vation of the vaccine
   6. For religious beliefs/reasons
   7. I don’t know where to get the vaccine
   8. I don’t know anything about the vac- cine

H. Others. (Please specify):

Thank you very much for partici- pating in this survey

Page 2
